# Salivary gland extract from the deer tick, *Ixodes scapularis*, facilitates neuroinvasion by Powassan virus in BALB/c mice

**DOI:** 10.1038/s41598-021-00021-2

**Published:** 2021-10-22

**Authors:** Rodrigo I. Santos, Meghan E. Hermance, Erin S. Reynolds, Saravanan Thangamani

**Affiliations:** 1 Department of Pathology, University of Texas Medical Branch, Galveston, Texas USA; 2 Department of Microbiology and Immunology, University of South Alabama College of Medicine, Mobile, Alabama USA; 3 Department of Microbiology and Immunology, SUNY Upstate Medical University, Syracuse, New York USA; 4 SUNY Center for Vector Borne Diseases, SUNY Upstate Medical University, Syracuse, New York USA; 5 Institute for Global Health and Translational Sciences, SUNY Upstate Medical University, Syracuse, New York USA

**Keywords:** Diseases, Medical research

## Abstract

Powassan virus (POWV) is a neuroinvasive flavivirus transmitted to mammals by the bite of ixodid ticks. In this study, we sought to investigate the impact of tick salivary gland extract (SGE) on POWV neuroinvasion. BALB/c mice were footpad inoculated with either a high dose or a low dose of POWV, with and without *Ixodes scapularis* salivary gland extract. Brain and spinal cord were extracted daily, and immunohistochemical techniques were used for temporal tracking of POWV antigen. The temporal pattern of POWV staining showed a caudal to rostral spread of POWV in the brains of mice from both high dose infection groups. For the high dose infection groups, the presence of tick SGE did not influence the spread of POWV in the brain. Mice infected with the low dose of virus alone did not present POWV staining in the brain; however, in the presence of SGE, low dose infected mice presented scattered foci of POWV-infected cells throughout the brain. This study shows that tick SGE facilitates POWV neuroinvasion when mice are infected with the lower dose of POWV. We also found two patterns of central nervous system invasion that were directly influenced by the dose of POWV administered.

## Introduction

Since 1958, when Powassan virus (POWV) was first isolated from the brain of a young boy who died of encephalitis in Powassan, Ontario^1^, we have known that this flavivirus is capable of causing a severe neuroinvasive disease in humans. The most common clinical presentations of disease caused by POWV are encephalitis, meningoencephalitis, and aseptic meningitis. The incubation period ranges from 1 to 5 weeks; however, most patients are unable to pinpoint the exact date of their exposure^2^. Lethargy, headache, sore throat, and disorientation often occur in the prodrome^3^. The encephalitic phase typically involves vomiting, prolonged fever, respiratory distress, loss of coordination, difficulty speaking, and seizures, ultimately resulting in a case fatality rate of approximately 10% and long-lasting neurological sequelae in 50% of survivors^2,4^. In several early POWV case studies, the neurological sequelae documented in individuals who survived POWV infection include hemiplegia, muscle wasting, recurrent severe headaches, and memory problems^3,5–9^. In more recent case series, encephalopathy is a commonly reported clinical syndrome typically used to describe overall brain dysfunction and altered mental status^10,11^.

Detailed pathological examination of postmortem tissues has been documented for several fatal cases, demonstrating that human cases of POWV encephalitis are typically characterized by perivascular and parenchymal infiltration of lymphocytes and monocytes, with occasional necrosis and neuronal loss^1,2,12,13^. Furthermore, the presence of POWV antigen is marked in neurons, suggesting that POWV has a strong neurotropism^1,12,13^. Additional information on the pathogenesis of POWV has been obtained through animal models. In rhesus macaques POWV displayed a marked neurotropism, with neurons and glial cells frequently being infected^14^. When 10^4^ PFU of POWV was intraperitoneally injected into 3–5-week-old BALB/c mice, the onset of clinical disease was between 5 to 6 days post-infection (dpi), with an average survival length of eight days and 100% mortality^15^. Similar observations were made for 6-week-old BALB/c mice infected by tick feeding^16^. In these mouse studies, signs of febrile illness (ruffled fur, malaise, and hunched posture) preceded the encephalitic phase of disease, which involved ataxia, hind limb paralysis, occasional convulsions, and death^15,16^. Histopathology of the brain sections from mice that succumbed to POWV infection demonstrated meningoencephalitis with focal mononuclear infiltrates and perivascular cuffing. Necrosis and POWV antigen were widely distributed throughout the cerebra of POWV-infected mice; however, the immunoreactive neurons were morphologically normal and were more prevalent than the inflammatory and necrotic lesions^15^.

POWV is a member of the Tick-borne encephalitis virus (TBEV) serogroup, and all viruses in this group are vectored by *Ixodes* species ticks. For an ixodid tick to successfully attach to a host and complete its lengthy feeding process, it must overcome the host’s immune and hemostatic defenses. Tick saliva contains an array of bioactive molecules that are vital to overcome the coagulation, hemostasis, wound healing, and innate and adaptive immune responses of the host^17–23^. In a process known as saliva-assisted transmission (SAT), the complex bioactive molecules present in tick saliva create a favorable environment for pathogen transmission and proliferation^24^. Experimental evidence of SAT is demonstrated when inoculation of the pathogen plus tick saliva into a host results in enhanced pathogen transmission and infection, compared to the levels of infection when the pathogen alone is inoculated^25^. Direct experimental evidence of SAT has been demonstrated for several tick-borne pathogens, including TBEV, which is closely related to POWV^24,26,27^. Although many reports support the concept of SAT for flavivirus pathogenesis^24,28–31^, other studies have shown no effect on viral pathogenesis due to arthropod saliva^32–34^. The reason behind these discrepancies are uncertain.

Our laboratory developed mouse models for POWV pathogenesis that resemble the course of disease for human cases of POWV^31,35^. These mouse models deliver virus via footpad inoculation, which penetrates through the dermis and reaches the subcutaneous layer of the skin. Thus, our models mimic the anatomical location of tick feeding while simultaneously allowing us to control the quantity of virus and tick saliva delivered to the host. In our first POWV neuropathological characterization, which was conducted with 4-week-old male C57BL/6 mice, we observed meningoencephalitis with perivascular mononuclear infiltration and microglial activation in the brain. A poliomyelitis-like syndrome with a high level of POWV antigen was detected at the ventral horn of the spinal cord, with motor neurons as major targets for POWV infection^35^. These findings from our C57BL/6 mouse model are similar to the histopathological descriptions observed in humans.

Our previous study showed that when *Ixodes scapularis* salivary gland extract (SGE) was co-inoculated with 10^3^ PFU of POWV, the tick SGE facilitates infection and influences disease outcome of 6-week-old female BALB/c mice^31^. This phenomenon was associated with enhanced POWV transmission and dissemination and decreased survival times for BALB/c mice infected with a lower dose (10^3^ PFU) of POWV plus tick SGE^31^. To investigate this phenomenon further, we undertook a temporal examination of the brain and spinal cord sections using histological techniques.

In the present study, we used the brain and spinal cord samples harvested from the BALB/c mice reported in our previous publication^31^; however, this study focuses on the histopathologic manifestations of POWV infection in the presence or absence of tick SGE. Here, our data clearly demonstrates that tick SGE facilitates POWV neuroinvasion when mice are infected with the lower dose of POWV. A caudal to rostral spread of POWV staining was detected in the brains of mice infected with the higher dose of POWV, regardless of the presence of SGE, while mice infected with the lower dose of POWV plus tick SGE displayed a scattered pattern of POWV staining throughout the brain.

## Methods

### Ethical statement

Animal experiments described in this study were conducted in accordance with an animal use protocol approved by the University of Texas Medical Branch (UTMB) Institutional Animal Care and Use Committee (IACUC; #0907054B),and ARRIVE guidelines.

### Samples

Tissue samples harvested from the mice reported in our earlier publication were used in this study^31^. Briefly, 5-week-old, female BALB/c mice were purchased from The Jackson Laboratory (Bar Harbor, ME), and were allowed to adapt to the local environment before being used in experiments, at which point the mice were approximately 6 weeks old.

Pathogen-free unfed *I. scapularis* adult females were used to generate SGE for our studies. All adult female *I. scapularis* were obtained from Oklahoma State University (OSU). The OSU colony of *I. scapularis* ticks is regularly tested for *Borrelia burgdorferi* and *Anaplasma phagocytophilum*, and all ticks used in our studies screened negative for these pathogens. Ticks were dissected using vannas spring scissors to cut the tick open and fine forceps to remove the salivary glands. A drop of sterile PBS was added to each tick when the dorsal flap was cut open. Each pair of tick salivary glands was immediately transferred to a new drop of sterile PBS on a glass slide. Salivary glands were rinsed in this second drop of PBS, and all non-salivary gland tissue debris was removed from the glands. The salivary glands were homogenized in a 1.5 mL microcentrifuge tube with a motorized pellet pestle, and the SGE was quantified using the microBCA protein assay kit (ThermoFisher Scientific).

Stock of POWV (LB strain), which was previously passaged 7 times in suckling mice brains, was passaged three times in Vero cells and the cell supernatant was harvested for use in the present studies. Each BALB/c mouse was infected under isoflurane anesthesia in the left hind footpad with 25 μL total of either (a) 10^6^ PFU of POWV plus 30 μg of SGE; (b) 10^6^ PFU of POWV alone; (c) 10^3^ PFU of POWV plus 30 μg of SGE; (d) 10^3^ PFU of POWV alone. The “2 SGE” notation in the figures represents SGE from two unfed adult *I. scapularis* females, which is equal to 30 μg^31^. Additionally, “lower dose” of POWV refers to mice infected with 10^3^ PFU of POWV, while the “higher dose” refers to mice infected with 10^6^ PFU of POWV. Mice from each treatment group were euthanized either at the following time points (n = 5 mice): 4, 5, 6, and 7 days post-infection (dpi), or as soon as the mice became moribund. At these time points, one brain hemisphere and a cervical vertebra were collected from every sacrificed mouse and formalin-fixed for 48 h.

### Immunohistochemistry

After 48 h of formalin fixation, the vertebrae were decalcified by treatment with 8% formic acid for 6 h. The brain and vertebrae tissues were dehydrated, embedded in paraffin, and sectioned (5 µm thickness) for histological studies. Slides were deparaffinized in xylenes and rehydrated in decreasing concentrations of ethanol. For visual referencing of brain areas, we used the Allen Institute’s open-access atlas of the mouse brain^36^. Representative histological images were organized according to regions/structures.

### POWV detection with the mouse-on-mouse reaction

The tissues were subjected to antigen retrieval with a citrate buffer target retrieval solution (Dako) for 20 min with microwave heating. Upon returning to room temperature, the slides were treated for endogenous peroxidase with a 30 min incubation in 6% H_2_O_2_. Mouse immune ascitic fluids (MIAF) were prepared against POWV (provided by Dr. Robert Tesh, UTMB). The Mouse-on-Mouse (MOM) kit (Vector Laboratories Inc.) was used to reduce the endogenous mouse IgG staining that can occur when mouse primary antibodies are used on mouse tissue^37,38^. Slides were incubated with the MOM kit blocking reagent for one hour at room temperature. The sections were washed, and then MIAF anti-POWV was diluted 1:300 in MOM kit diluent and incubated for 30 min at room temperature. MOM biotinylated anti-mouse IgG reagent was used as a secondary antibody and incubated at room temperature for 60 min. Detection of the biotinylated secondary antibody was performed with a streptavidin-peroxidase polymer (Sigma-Aldrich) followed by staining with the NovaRED HRP substrate kit (Vector Laboratories Inc.). Slides were counterstained with Harris hematoxylin and coverslips were mounted using Permount (ThermoFisher Scientific). Brain and vertebrae sections from uninfected mice were used as negative controls to verify the specificity of the MIAF anti-POWV primary antibody. Secondary antibody only (no primary antibody) controls were used to confirm that the MOM biotinylated anti-mouse IgG reagent did not bind non-specifically to cellular components^35,39^.

### Immunohistochemistry for CD11b detection

CD11b is a general marker present in all myeloid cell lineages^40^. For CD11b staining, selected slides were subjected to alkaline antigen retrieval with 10 mM Tris solution and 0.05% Tween 20 (pH 10) for 15 min with microwave heating^41^. Endogenous peroxidase quenching was executed as described in the previous section. Slides were blocked for 60 min in 5% goat serum followed by incubation with rabbit anti-CD11b (Abcam) diluted 1:100 and incubated for 1 h at 37 °C. The secondary antibody was a biotinylated goat anti-rabbit immunoglobulin (Dako) diluted 1:500 and incubated for 30 min at room temperature^42^. The biotin reaction, staining, and counterstaining were executed as described in the previous section. All serum and antibodies were diluted in PBS with 1% BSA and 0.1% Triton X-100, pH 7.4.

## Results

**Figure 1:**
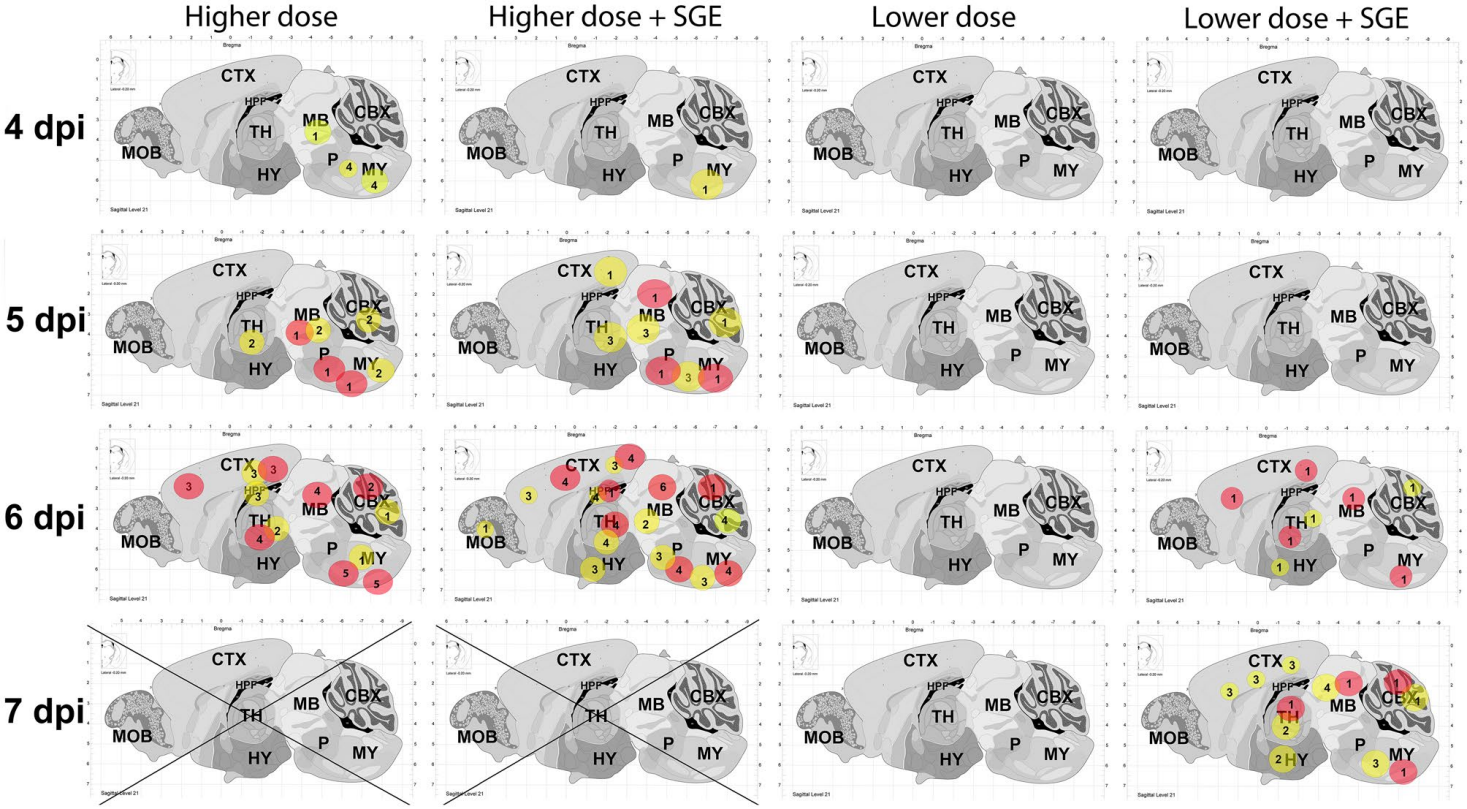
Schematic of POWV infection in the brains of BALB/c mice. Each column represents one treatment group. Lower dose of POWV refers to mice infected with 10^3^ PFU of POWV, and higher dose refers to mice infected with 10^6^ PFU of POWV. SGE indicated that 30 μg of *Ixodes scapularis* salivary gland extract was mixed to the virus before inoculum. Circles indicate POWV-infected regions of the brain. Red circle = highly infected region. Yellow circle = lowly infected region. The number inside each circle represents the number of mice with detectable POWV in a specific region of the brain. “X” represents time points at which all mice succumbed to disease. *MY* medulla, *P* pons, *MB* midbrain, *CBX* cerebellum, *TH* thalamus, *HY* hypothalamus, *HPF* hippocampal formation, *CTX* cortex, *MOB* main olfactory bulb.

**Figure 2:**
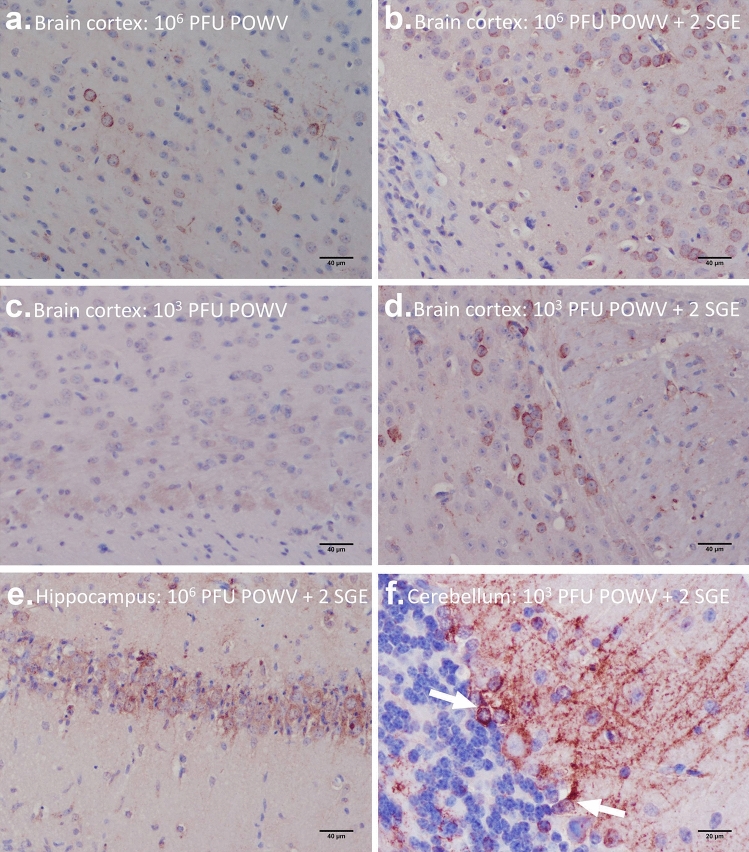
Immunohistochemistry for POWV detection in the brain. (**a**) 10^6^ PFU POWV, brain cortex. (**b**) 10^6^ PFU POWV + 2 SGE, brain cortex. (**c**) 10^3^ PFU POWV, brain cortex. (**d**) 10^3^ PFU POWV + 2 SGE, brain cortex. (**e**) 10^6^ PFU POWV + 2 SGE, hippocampus. (**F**) 10^3^ PFU POWV + 2 SGE, cerebellum. (**a**–**e**) Scale bars represent 40 µm. (**f**) Scale bars represent 20 µm. Arrows point to Purkinje cells.

Immmunohistochemical analysis detected POWV in the brains of mice in both higher dose treatment groups between 4 to 5 dpi, with the medulla and pons being the first regions to display POWV antigen. As 5 dpi progressed to 6 dpi, a caudal to rostral pattern of infection was observed (Fig. 1; Supplementary Table S1). The POWV-infected areas of the brain advanced from the brainstem, to adjacent brain regions, and ultimately to more superficial regions such as the cortex (Fig. 2a,b). As the encephalitis progressed and mice in both high dose groups became moribund at 6 dpi, focal POWV antigen was detected in brain regions distal to the brainstem (Fig. 1). Regardless of the presence or absence of SGE, all mice infected with high dose succumbed before 6 dpi. Overall, there was no difference in the pattern of brain infection when mice were infected with the high dose of POWV plus SGE, or with the high dose of POWV alone.

On the other hand, at lower doses of POWV, the presence of SGE appears to facilitate POWV neuroinvasion. POWV viral antigen was not detected in any histological section of the brain for the group of mice infected with the lower dose of POWV without SGE, and all mice survived to the time of study completion (11 dpi) (Figs. 1, 2c); however, when SGE was administered with the lower dose of POWV, diffuse areas of POWV infection were detected between 6 to 7 dpi, and all mice succumbed by 8 dpi (Fig. 1; Supplementary Table S2). The medulla, pons, cerebellum, midbrain, thalamus, hypothalamus, and cortex all displayed viral antigen at 6 and 7 dpi when mice were infected with the lower dose of POWV plus SGE (Figs. 1, 2d,f). These results suggest that at low doses of POWV, SGE plays a central role in facilitating POWV infection of the brain.

POWV antigen was detected in both the hippocampus and cerebellum of subjects that presented with diffuse brain infection (Fig. 2e,f). Mice that were infected with a higher dose of POWV, in the presence and absence of SGE, as well as mice infected with the low dose of POWV plus SGE, all displayed similar patterns of POWV infection in the hippocampus and cerebellum. When POWV antigen was detected in the cerebellum, the Purkinje cells were the most commonly infected cerebellar cell type (Fig. 2f).

**Figure 3:**
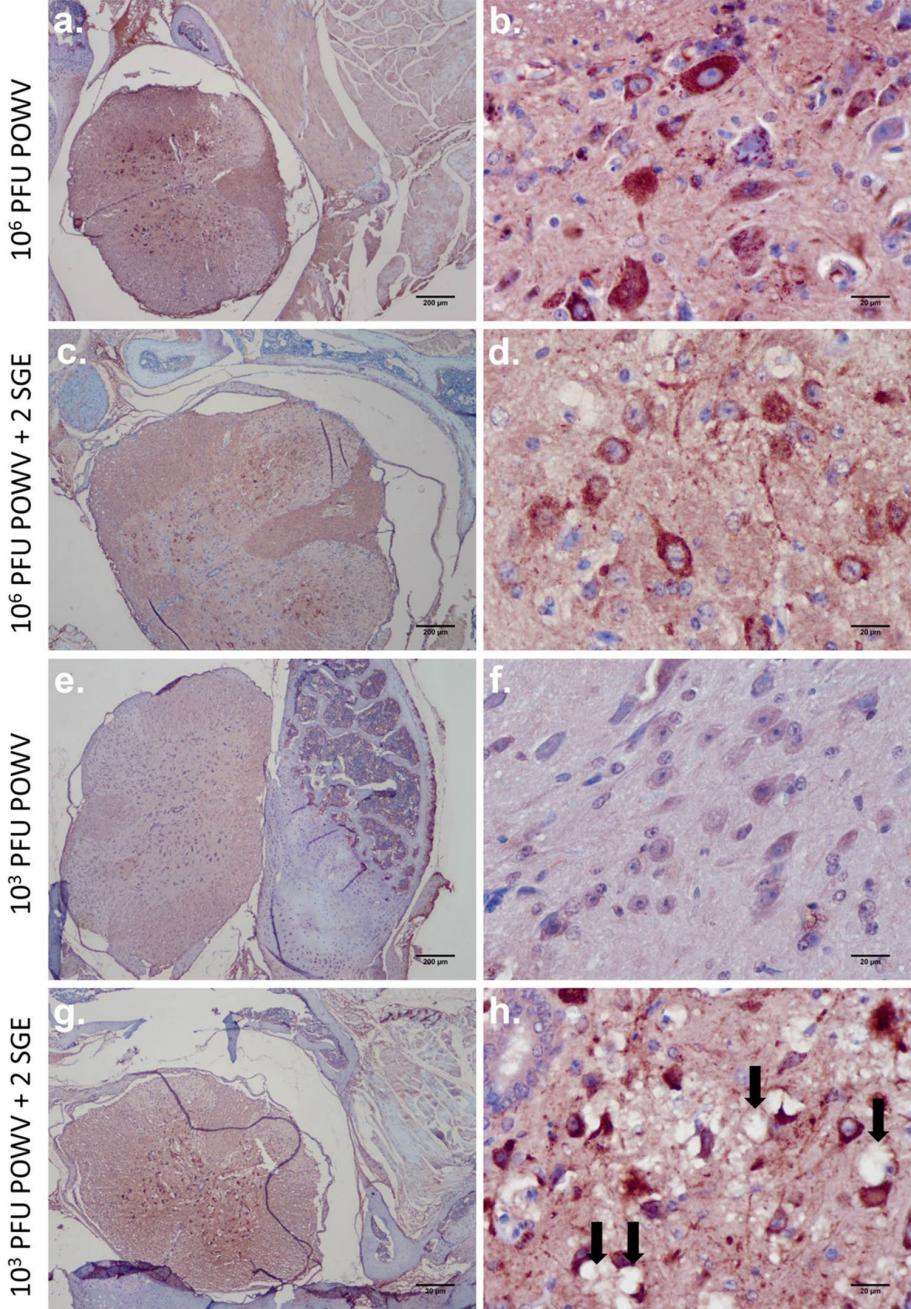
Immunohistochemistry for POWV detection in the spinal cord. (**a**,**b**) 10^6^ PFU POWV. (**c**,**d**) 10^6^ PFU POWV + 2 SGE. (**e**,**f**) 10^3^ PFU POWV. (**g**,**h**) 10^3^ PFU POWV + 2 SGE. Scale bars represent either 200 µm or 20 µm. Black arrows indicate neuropil vacuolization.

Spinal cord infection was consistently present in all groups of mice that displayed POWV antigen in the brain with the exception of one animal infected with low dose in the presence of SGE (Supplementary Table S2, 6 dpi with SGE). In both the presence and absence of SGE, POWV antigen was diffusely distributed throughout the spinal cords of mice infected with the high dose of POWV (Fig. 3a–d). A similar pattern of widespread infection throughout the spinal cord also occurred in the majority of mice infected with the lower dose of POWV plus SGE (Fig. 3g,h). Typical neurologic signs of infection including weak grip, ataxia, loss of balance, and hind limb paralysis followed by total paralysis, were directly correlated to the groups of mice that displayed POWV antigen in both the brain and spinal cord. For these mice, the presence of viral antigen in the spinal cord was widespread, with significant involvement of motor neurons. Neuropil vacuolation was detected in moribund subjects that were infected with the low dose of POWV plus SGE (Fig. 3g,h). With the exception of one mouse, POWV antigen was not detected in the spinal cord sections of mice infected with the low dose of POWV in the absence of SGE (Fig. 3e,f). The one mouse in the lower dose treatment group that displayed viral antigen presented two POWV-positive cells in the spinal cord (Supplementary Fig. S1; Supplementary Table S2).

By combining the immunohistochemistry data for POWV detection in the brain and the spinal cord, the early stages of POWV infection in the central nervous system (CNS) can be compared across the four treatment groups. These comparisons will help to generate a timeline of POWV neuroinvasion. Supplementary Tables S1 and S2 and Figs. 1, 2, and 3 depict the different patterns of early CNS infection among the treatment groups of mice. Mice infected with the higher dose of POWV, in the absence of SGE, typically displayed well-defined foci of POWV antigen in the spinal cord during early CNS infection (Fig. 3a,b), and the medulla and pons were the only regions of the brain where viral antigen was detected at this early stage of CNS infection (Fig. 1; Supplementary Table S1). In the group of mice infected with the higher dose of POWV plus SGE, features were similar to the early stages of CNS infection in the no-SGE higher dose group, where well-defined foci of POWV-positive cells appear in the spinal cord and in caudal regions of the brain (Figs. 1, 3c,d). The earliest immunohistochemical detection of POWV antigen in the CNS occurred at 4 dpi for both of these higher dose treatment groups of mice. At the lower dose of infection in the absence of SGE, no POWV antigen was detected in sections of the spinal cord or brain (Figs. 1, 2c, 3e,f), with the exception of one mouse where POWV antigens were detected in the spinal cord (Supplementary Fig. S1; Supplementary Table S2). For mice infected with the lower dose of POWV plus SGE the earliest immunohistochemical detection of viral antigen in the CNS occurred at 6 dpi. This neuroinvasion timeframe was delayed by two days when compared to the mice infected with the higher dose of POWV.

**Figure 4:**
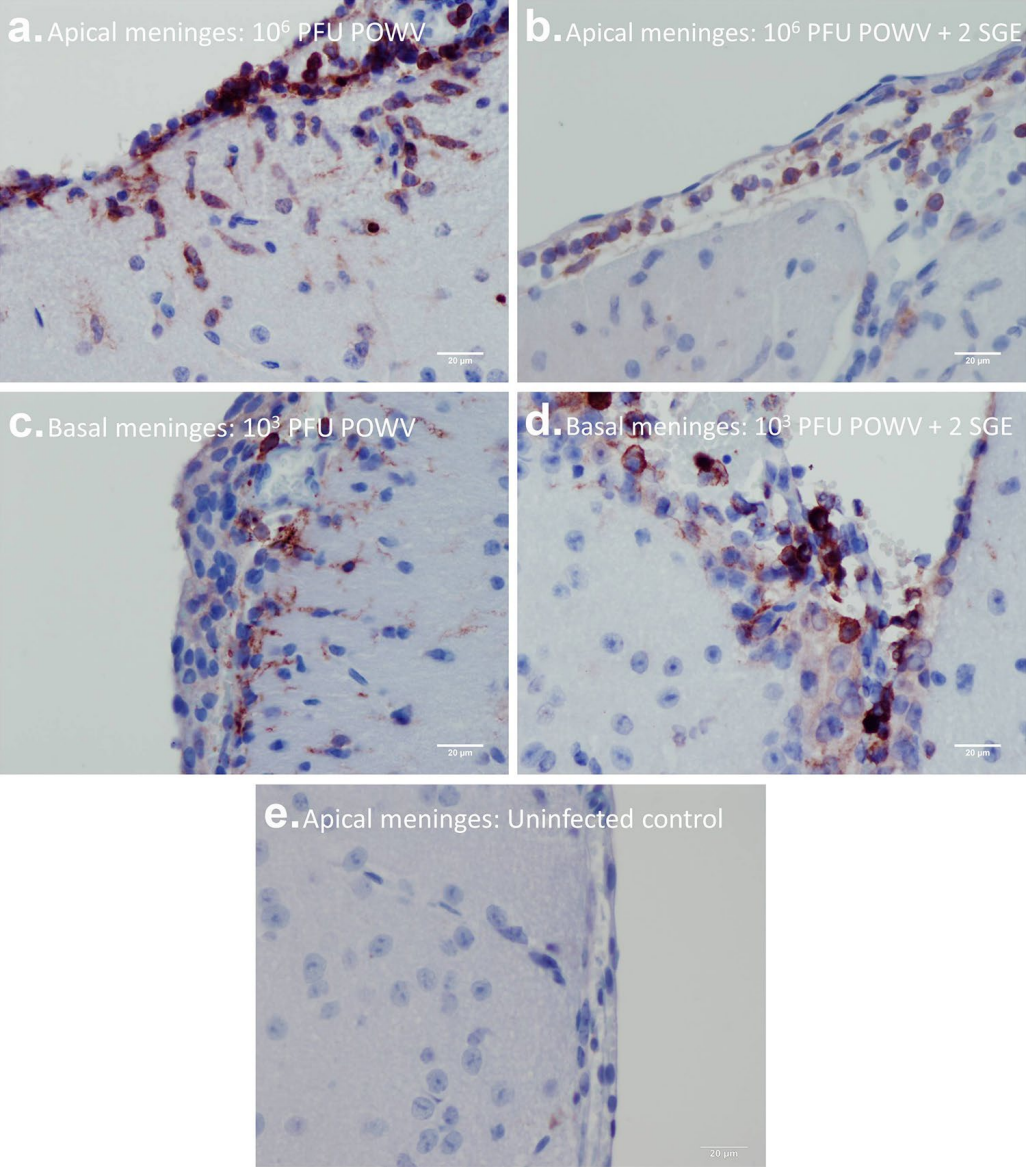
Immunohistochemistry for CD11b detection in the brain. (**a**) 10^6^ PFU POWV, apical meninges. (**b**) 10^6^ PFU POWV + 2 SGE, apical meninges. (**c**) 10^3^ PFU POWV, basal meninges. (**d**) 10^3^ PFU POWV + 2 SGE, basal meninges. (**e**) Uninfected control, apical meninges. Scale bars represent 20 µm.

For mice in all four treatment groups, infiltration of mononuclear cells to the meninges occurred before POWV antigen was detected in the CNS. CD11b is a common marker present in myeloid lineage cells^40^; therefore, it was used in this study to detect inflammatory cell infiltrates in the meninges. The majority of mice in this study presented with CD11b-positive cells in the meninges as early as 3 dpi. As POWV infection of the CNS progressed, moribund mice displayed widespread meningitis throughout the brain, including in the apical meninges (Fig. 4a,b). In the low dose treatment groups, both with and without SGE, CD11b-positive cells were more abundant in the basal meninges than the apical meninges (Fig. 4c–e). At the early stages of CNS involvement, this pattern of basilar meningitis was also detected in mice from both high dose treatment groups. Although viral antigen was not detected in the brain sections of mice infected with the low dose of POWV in the absence of SGE, mice in this treatment group displayed a mild meningitis (Fig. 4c).

## Discussion

In the present study, we demonstrated that tick SGE generated from unfed female *I. scapularis* potentiates POWV neuroinvasiveness when POWV is administered at a lower dose (10^3^ PFU). We included the 10^3^ PFU dose in this study as it is representative of the flavivirus titer expected per tick based on previous studies. Unfed female *I. scapularis* ticks were used to generate SGE for this study because POWV can be transmitted as early as 15 min after initiation of tick feeding^16,43^. SGE derived from partially fed or fully fed ticks would not accurately represent this early timeline of tick-borne flavivirus transmission. On the other hand, we used 10^6^ PFU to better understand the effect of tick SGE under an extreme condition.

POWV viral antigen was not detected in any brain section for the group of mice infected with the lower dose of POWV in the absence of SGE. Unexpectedly, we also found two neuroinvasion patterns depending on the viral dose administered. When a lower dose of POWV plus SGE was administered, the presence of POWV antigen in the brain occurred abruptly at 6 or 7 dpi, with scattered foci of infected cells throughout the brain, suggesting that a hematogenous route of neuroinvasion may occur for the mice infected with the low dose of POWV plus SGE. For the higher dose (10^6^ PFU) inoculum, animals presented a caudal to rostral pattern of brain infection, regardless of the presence or absence of SGE. Caudal to rostral dissemination of POWV in the brain is suggestive of the neuronal route of CNS invasion; however, this hypothesis was drawn based on only the immunohistochemical assays conducted in the present study. Further experiments with more sensitive techniques are necessary to determine whether the hypothesis is supported.

Our previous study showed that there was no difference in disease outcome or virus dissemination when mice were infected with the higher dose of POWV plus SGE, or with the higher dose of POWV alone. All mice in the higher dose treatment groups displayed total paralysis and ultimately succumbed to disease at 6 dpi. There were no significant differences between brain viral loads for mice in the group that received the higher dose of POWV versus the mice that received the higher dose plus SGE^31^. Thus, we hypothesized that when a higher dose of POWV is administered in the presence of SGE, any potential effect that SGE has on POWV dissemination and course of disease is saturated by the high virus titer. For mice inoculated with the lower dose of POWV in the presence of tick SGE, we observed enhanced POWV transmission and dissemination, accelerated disease progression, and decreased survival times compared to mice inoculated with the virus alone. All mice infected with the lower dose of POWV plus SGE succumbed to disease between 7 and 8 dpi; however, the mice infected with the lower dose of POWV in the absence of SGE failed to display clinical signs of infection and all survived to the study termination (11 dpi)^31^. The current study addresses the temporal histopathological aspects of the POWV infection executed in the previous publication.

Arthropod saliva can enhance transmission of pathogens. The mechanisms that drive this enhancement are not clear, but it is speculated that it is related to the immune modulation at the feeding site of the vector^44^. The first observation of saliva potentiation of pathogen transmission was with *Leishmania*. *Leishmania major* lesions increase in size and number when the parasites are inoculated in the presence of *Lutzomia* species SGE^45^. Later, many publications on arthropod-borne pathogens demonstrated saliva-dependent infection enhancement, or SAT. For example, Cache Valley virus had higher infectivity when uninfected mosquitoes had fed before infection of the virus by needle^46^. Hajnicka et al. demonstrated that *Dermacentor reticulatus* SGE promotes Vesicular Stomatitis virus replication by suppressing the effect of Interferon type I^47^. Additional evidence for SAT was found in La Crosse virus and *Orientia tsutsugamushi* studies^48,49^. Within the Flaviviridae family there are some conflicting data about SAT importance for viral pathogenesis. Mice previously subjected to *Aedes aegypti* probing and then infected with Dengue virus presented higher viremia than mice not fed on by mosquitoes^50^. Furthermore, West Nile virus infections via mosquito feeding had higher blood and tissue titers and faster neuroinvasion than WNV infections via needle inoculation^28,51^. Yet, some reports have shown no difference comparing animals infected in the presence or absence of the vector arthropod saliva^32–34,52^.

To date, the mechanism(s) by which neurotropic tick-borne flaviviruses invade the CNS is not clear^53^. Evidence of lymph node infection six hours after subcutaneous inoculation of TBEV, and the subsequent primary viremia, caused many researchers to believe that viruses in the TBEV serogroup invade the CNS via the hematogenous route^54,55^. On the other hand, studies showing virus infection of the olfactory bulb have suggested that retrograde neuronal invasion^56^ is a mode of CNS invasion for members of TBEV complex^57,58^, Saint Louis Encephalitis virus^59^, WNV^59^, Venezuelan Equine Encephalitis virus^60^, and Murray Valley Encephalitis virus^61^. The present study is unique because it is the first to show a dose-dependency for the pattern of neuroinvasiveness in the *Flaviviridae* family.

## Conclusions

This study adds important knowledge on flavivirus neuropathogenesis. We used immunohistochemical techniques for temporal tracking of POWV antigen throughout the CNS. These experiments show that at low doses of POWV, the presence of *I. scapularis* SGE facilitates POWV neuroinvasion. We found two patterns of CNS invasion that were directly influenced by the dose of virus administered. The first pattern observed is consistent with neuronal spread of the virus and was present in mice infected with the higher dose (10^6^ PFU) of POWV. These mice displayed a gradual caudal to rostral spread of POWV in the brain, which was not facilitated by the presence of tick SGE. The second pattern of CNS invasion was consistent with hematogenous spread of the virus, and the presence of tick SGE facilitated this pattern of CNS invasion for mice infected with the lower dose (10^3^ PFU) of POWV. For this pattern, POWV antigen appeared abruptly, with scattered foci of infected cells throughout the brain. Our data suggests that POWV might have the ability to reach the brain by either the neuronal or hematogenous route. The neuronal route would require high levels of virus, while the hematogenous route could occur at virus titers similar to what is observed in the natural infection by ticks. We cannot rule out the possibility for dose-dependent neuroinvasiveness for other flaviviruses. This could elucidate some of the conflicting data about SAT and neuroinvasiveness present in the *Flaviviridae* literature.

## Supplementary Information

Supplementary Figure S1.

Supplementary Table S1.

Supplementary Table S2.
