# Supplementary figures and images for: Salivary gland extract from the deer tick, Ixodes scapularis, facilitates neuroinvasion by Powassan virus in BALB/c mice
Source: Sci Rep. 2021 Oct 22;11:20873. doi: 10.1038/s41598-021-00021-2 (PMC8536725; doi:10.1038/s41598-021-00021-2)

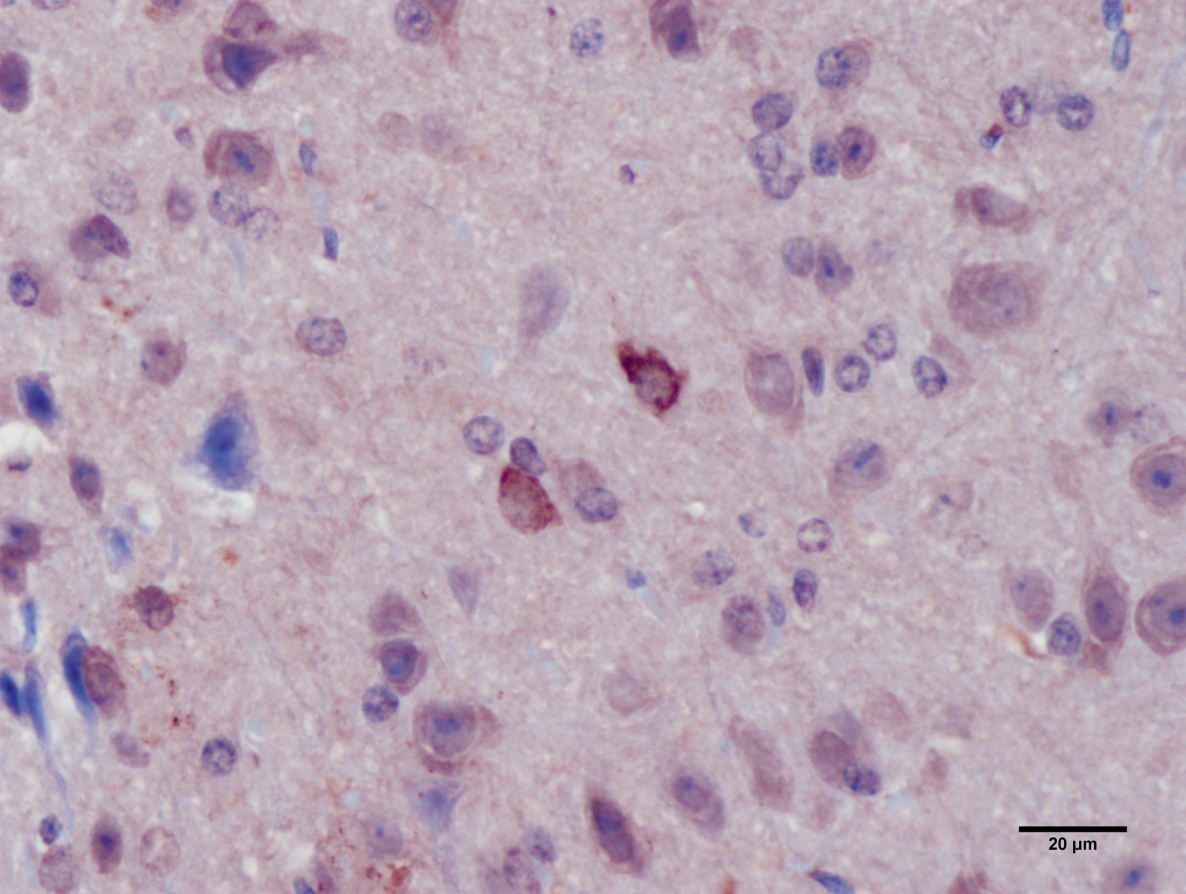

Supplement: Supplementary file 1 — Supplementary Figure S1. [file 41598_2021_21_MOESM1_ESM.jpg]
