# Supplementary material for: Salivary gland extract from the deer tick, Ixodes scapularis, facilitates neuroinvasion by Powassan virus in BALB/c mice
Source: Sci Rep. 2021 Oct 22;11:20873. doi: 10.1038/s41598-021-00021-2 (PMC8536725; doi:10.1038/s41598-021-00021-2)
Supplement: Supplementary file 3 — Supplementary Table S2. [file 41598_2021_21_MOESM3_ESM.doc]

|  | | 4dpi | | | | | 4dpi | | | | | 5dpi | | | | | 5dpi | | | | | 6dpi | | | | | 6dpi | | | | | 7dpi | | | | | 7dpi | | | | |
| --- | --- | --- | --- | --- | --- | --- | --- | --- | --- | --- | --- | --- | --- | --- | --- | --- | --- | --- | --- | --- | --- | --- | --- | --- | --- | --- | --- | --- | --- | --- | --- | --- | --- | --- | --- | --- | --- | --- | --- | --- | --- |
|  | | Media | | | | | SGE | | | | | Media | | | | | SGE | | | | | Media | | | | | SGE | | | | | Media | | | | | SGE | | | | |
| ihc | Spinal cord |  |  |  |  |  |  |  |  |  |  |  |  |  |  |  |  |  |  |  |  |  |  |  |  |  |  | N |  |  |  |  |  |  |  |  |  |  | N |  |  |
|  | Cerebellum |  |  |  |  |  |  |  |  |  |  |  |  |  |  |  |  |  |  |  |  |  |  |  |  |  |  |  |  |  |  |  |  |  |  |  |  |  |  |  |  |
|  | Medulla/Pons |  |  |  |  |  |  |  |  |  |  |  |  |  |  |  |  |  |  |  |  |  |  |  |  |  |  |  |  |  |  |  |  |  |  |  |  |  |  |  |  |
|  | Midbrain |  |  |  |  |  |  |  |  |  |  |  |  |  |  |  |  |  |  |  |  |  |  |  |  |  |  |  |  |  |  |  |  |  |  |  |  |  |  |  |  |
|  | Thalamus |  |  |  |  |  |  |  |  |  |  |  |  |  |  |  |  |  |  |  |  |  |  |  |  |  |  |  |  |  |  |  |  |  |  |  |  |  |  |  |  |
|  | Hypothalamus |  |  |  |  |  |  |  |  |  |  |  |  |  |  |  |  |  |  |  |  |  |  |  |  |  |  |  |  |  |  |  |  |  |  |  |  |  |  |  |  |
|  | Hippocampus |  |  |  |  |  |  |  |  |  |  |  |  |  |  |  |  |  |  |  |  |  |  |  |  |  |  |  |  |  |  |  |  |  |  |  |  |  |  |  |  |
|  | Cortex |  |  |  |  |  |  |  |  |  |  |  |  |  |  |  |  |  |  |  |  |  |  |  |  |  |  |  |  |  |  |  |  |  |  |  |  |  |  |  |  |

Table S2. Summary of POWV staining in the Central Nervous System for low dose infected animals

Results shown are POWV staining comparisons between the treatment groups. Each column represents a single mouse. White boxes represent brain regions with no POWV-positive cells. The colors in each box represent the intensity of POWV-positive staining for each brain region, where the yellow represents low levels of POWV staining and the black represents widespread/intense POWV staining. The color ranking from low to high intensity are: . “N” means not available.

#### Figure S1: Immunohistochemistry for POWV detection in the spinal cord.

This is the only subject in the 10^3^ PFU POWV treatment group that displayed a POWV-positive cell.
